# Delirium prevention in hospices: Opportunities and limitations – A focused ethnography

**DOI:** 10.1177/02692163241310762

**Published:** 2025-01-21

**Authors:** Imogen Featherstone, Miriam J Johnson, Trevor Sheldon, Rachael Kelley, Rebecca Hawkins, Alison Bravington, Sarah Callin, Rachael Dixon, George Obita, Najma Siddiqi

**Affiliations:** 1 Department of Health Sciences, University of York, York, UK; 2 Wolfson Palliative Care Research Centre, Hull York Medical School, University of Hull, Hull, UK; 3 Wolfson Institute of Population Health, Barts and The London School of Medicine and Dentistry, Queen Mary University, London, UK; 4 School of Health, Leeds Beckett University, Leeds, UK; 5 Leeds Institute of Health Sciences, University of Leeds, Leeds, UK; 6 Saint Catherine’s Hospice, Scarborough, UK; 7 Dove House Hospice, Hull, UK; 8 Hull York Medical School, University of York, York, UK; 9 Bradford District Care Trust, Bradford, UK

**Keywords:** Delirium, qualitative research, palliative care, hospices

## Abstract

**Background:**

Delirium is common and distressing for hospice in-patients. Hospital-based research shows delirium may be prevented by targeting its risk factors. Many preventative strategies address patients’ fundamental care needs. However, there is little research regarding how interventions need to be tailored to the in-patient hospice setting.

**Aim:**

To explore the behaviours of hospice in-patient staff in relation to delirium prevention, and the influences that shape these behaviours.

**Design:**

Focused ethnography supported by behaviour change theory. Observation, semi-structured interviews and document review were conducted.

**Setting/participants:**

A total of 89 participants (multidisciplinary staff, volunteers, patients and relatives) at two UK in-patient hospice units.

**Results:**

Hospice clinicians engaged in many behaviours associated with prevention of delirium as part of person-centred fundamental care, without delirium prevention as an explicit aim. Carrying out essential care tasks was highly valued and supported by adequate staffing levels, multidisciplinary team engagement and role clarity. Patients’ reduced physical capability limited some delirium prevention behaviours, as did clinicians’ behavioural norms related to prioritising patient comfort. Delirium prevention was not embedded into routine assessment and care decision-making, despite its potential to reduce patient distress.

**Conclusions:**

The value placed on fundamental care in hospices supports delirium prevention behaviours but these require adaptation as patients become closer to death. There is a need to increase clinicians’ understanding of the potential for delirium prevention to reduce patient distress during illness progression; to support inclusion of delirium prevention in making decisions about care; and to embed routine review of delirium risk factors in practice.

**What is already known about the topic?**

- Delirium is common and distressing for hospice in-patients.
- Hospital-based research shows delirium can be prevented by targeting its risk factors.
- Research is needed on how interventions need to be tailored to the in-patient hospice setting.

**What this paper adds?**

- Hospice culture and organisation supports clinicians to engage in many behaviours that are preventative for delirium as part of person-centred fundamental care.
- Delirium prevention is not embedded into routine assessment and care decision-making.
- Delirium prevention behaviours are limited, not only due to patient’s illness progression, but also clinicians’ behavioural norm of ‘wrapping patients up’ to care for them.

**Implications for practice, theory or policy**

- Delirium prevention is supported by an organisational culture which values fundamental care, adequate staffing and multidisciplinary team engagement.
- Behaviour change intervention is needed to increase clinicians’ understanding of the potential to reduce patient distress by continuing delirium prevention behaviours during illness progression and to support them to include prevention in routine assessment and care decision-making.

## Introduction

Delirium is commonly experienced by hospice in-patients^1^ and is distressing for patients, their families and clinicians.^2^ It is characterised by acute, fluctuating disturbances in attention, awareness and cognition and has underlying physiological causes.^3^

Systematic reviews of delirium prevention interventions targeting risk factors in hospital settings report a reduced incidence of between a third and a half.^4–6^ Interventions typically address patients’ care needs including orientation, sleep, sensory needs, hydration, nutrition, bladder and bowel function, infection, hypoxia, pain and medication management. This closely aligns with ‘fundamental care’, meeting patients’ essential care needs through person-centred interaction.^7^ In hospital settings, recurrent deficits in meeting these needs, including eating, drinking and mobilising, have been reported.^8^ Researchers have highlighted that delirium prevention could be a valuable driver to improve broader quality of care.^4,9–12^

In the UK context, hospices are mostly independent, charitably funded organisations which provide in-patient symptom management and end-of-life care, as well as other palliative care services. Palliative care patients are at particular risk of delirium due to factors including their illness severity and use of high-risk medications.^13^ Due to the high prevalence of delirium in palliative care patients,^1^ it is sometimes assumed that delirium is non-preventable in this setting, particularly close to the end of life.^14^ However, preventative interventions in other clinical settings where delirium was once thought unavoidable, such as ICU, *have* reduced its incidence.^15^ Furthermore, cultural and organisational factors including person-centred care, teamworking and adequate staffing, may support delirium prevention strategies in hospices.^16^ However, delirium prevention strategies may need to be tailored to hospices and adapted as patients’ illness progresses.^17^

There has been little delirium prevention research in palliative care settings. A qualitative systematic review found no delirium prevention studies;^2^ there is a need for further non-pharmacological prevention trials in palliative care settings.^18^ A minimal intervention study, involving re-orientation and medication review, found no reduction in delirium incidence.^19^ A pilot trial of a prevention intervention in palliative care units reported low adherence but also a signal of benefit.^14^

In a qualitative interview study,^16^ informed by behaviour change theory,^20^ we found that hospice clinicians’ practice focused on delirium *management*, rather than prevention. Clinicians’ emotional responses to delirium-related distress were a powerful driver of their practice, so behaviour change techniques which increase their understanding of the potential for prevention to reduce delirium-related distress may be effective. In these interviews, clinicians may not have reported carrying out tasks that support delirium prevention, delivered as part of usual care, due to lack of awareness of their relevance. Building upon these findings, we conducted a focused ethnography using observation to explore the nature and extent of delirium prevention behaviours during routine care to inform the development of tailored delirium prevention interventions for hospice settings.

### Study aim

To explore the behaviours of hospice in-patient staff in relation to delirium prevention, and the influences that shape these behaviours.

## Methods

We report this study according to the Standards for Reporting Qualitative Research (SRQR).^21^

### Theory and methodology

This study took an ethnographic approach to explore culture, perspectives and practices in the hospice context in relation to delirium prevention behaviours.^22^ We used focused ethnography which is more selective, time-limited and problem-focused than traditional ethnography.^23^

We used behaviour change theory including the COM-B framework (Capability, Opportunity, Motivation and Behaviour) and Mechanisms of Action (MoAs; Supplemental File 1) to enable detailed analysis of influences on hospice staff’s delirium prevention behaviours.^20,24^

We took a critical realist stance, based on the assumption of a shared reality, our understanding of which is mediated by our cultural contexts.^25^

#### Setting

The in-patient units at two UK hospices which provide symptom management and end-of-life care were included. Most in-patients have a primary diagnosis of cancer but they also provide care for patients with other life-limiting illnesses. The two hospices are run independently by different charitable organisations. As COVID-19 restrictions remained in place during the observation period, the number of patient beds available were reduced (H1 = 11 beds and H2 = 8 beds) and all rooms were single occupancy (H1 usually has some shared rooms). There were also restrictions to visits by family and friends.

#### Population

Eligible participants were staff and volunteers involved in the care of patients on the hospice in-patient units; adult (>18 years) hospice in-patients and their relatives. Patients and relatives who, in the opinion of hospice staff, were too distressed to be approached about participation, were not included in the study.

#### Sampling

The in-patient units at two hospices were the cases selected for this study, enabling comparison of the influence of organisational culture and environment on care. Purposive sampling was used to include different roles involved in patient care (doctors, nurses, therapists, health care assistants (HCAs), catering staff and volunteers) as well as patients and their families. Observation was planned to include different contexts of care and communication by the multidisciplinary team for example, patients’ rooms, nurses’ stations, staff offices, handovers and team meetings. Data sufficiency was determined when repeated patterns of behaviour and behavioural influences had been observed.^26^

#### Recruitment

The consent approach was developed with public involvement (PI) group members with experience of delirium or hospice care. Staff and volunteers were initially informed about the study through posters displayed on the units and staff meetings. A hospice doctor made the initial approach to patients about the research study. All potential participants were provided with an information leaflet and the researcher (IF) explained the study as clearly and simply as possible. Written informed consent was gained from participants. This included whether patients wished to continue to participate if they were to lose capacity in the future. If a patient did not have capacity to consent, a personal consultee was asked for advice on their participation and to complete a consultee declaration form. The researcher (IF) took an ongoing, reflexive approach to consent: checking the acceptability of continuing observations with patients, family and clinicians as patients’ illnesses progressed and reducing observation of patient care when it was sensitive to do so.^27,28^

### Data collection

The researcher (IF) shadowed staff during care of consenting patients in different contexts. She talked informally with participants to explore influences on observed behaviours. In negotiating relationships with participants, she aimed to balance closeness to gain nuanced understandings of their perspectives with retaining some distance to enable a critical, analytic perspective.^29,30^ Brief fieldnotes were made and written up fully, shortly after observations, to optimise recall.

An observation guide (Supplemental File 2) was developed using delirium prevention behaviours from clinical guidelines,^31–33^ reviewed for relevance to hospices by an expert panel (clinicians and researchers) and PI members and COM-B prompts to explore influences on observed behaviours.^20^ These included: risk factor review, including medication; pain management; addressing cognitive impairment, sensory needs, hypoxia and infection; sleep routines; optimising mobility, hydration, nutrition and bladder and bowel function. Observations included how routinely these were carried out and by whom. COVID-19-related restrictions affected some aspects of practice observed.

Hospice clinical records of consenting patients were examined for documentation of delirium prevention practices. This enabled data collection to include periods of care that had not been observed, care processes and team communication.

Following the observation period at each hospice, semi-structured interviews, informed by COM-B and MoAs,^20,24^ were conducted with clinicians to clarify and extend understanding of influences on observed behaviours. This included exploring clinicians’ understanding of delirium prevention; their intentions when carrying out preventative tasks during routine care; supportive factors and limitations in the hospice setting. Interviews were recorded and encrypted using an audio recorder, and transcribed verbatim.

### Data analysis

Analysis began during the observation period using an iterative approach. The researcher (IF) reflected on and discussed observed behaviours and influences with the wider team (MJ, NS) and wrote analytic memos which focused further observations and discussion with participants.^30^

We used thematic analysis,^34^ informed by Fryer’s^35^ critical realist approach, in that we sought to develop explanatory understanding of the mechanisms influencing observed delirium prevention behaviours. Coding was supported by Nvivo software.^36^ Inductive open-coding was used to explore and develop codes, followed by development of summary statements of influences for each preventative behaviour, coded to MoA and COM-B concepts and presented in tables (IF)^37,38^ (e.g. table: Optimising mobility, Supplemental File 3).

This enabled analysis of both influences on specific delirium prevention behaviours and those that are cross-cutting across several behaviours. We used the Theory and Techniques tool^39^ to identify behaviour change techniques (BCTs) to target these influences in an intervention. The developing analysis was regularly reviewed by research team members (MJ, AB, NS and TS) and the PI group.

### Ethical approval

This study was approved by the NHS Bradford Leeds Research Ethics Committee (Ref: 19/YH/0323; 02/12/19) and hospice institutional permissions gained.

## Results

Eighty-nine participants were included in the study including sixty-two hospice staff; two volunteers and two family members. Twenty-three patients participated: twenty-one had capacity to consent and two required declarations from personal consultees. Please see Table 1 below for further participant details. No participants withdrew from the study.

**Table 1.** Participant characteristics.

Participants	Number
Patients	Total = 23 Hospice 1 = 12 Hospice 2 = 11
Sex	Male = 11 Female = 12
Age	Mean = 73.13 years Range = 45–97 years
Primary diagnosis	Cancer = 21 Other = 2
Family members	2
Volunteers	2
Hospice staff	Total = 62 Hospice 1 = 36 Hospice 2 = 26
Sex	Male = 10 Female = 52
Nurses	23
Health care assistants	15
Doctors	15
Therapists (physio, occupational, music and complementary therapy)	7
Social worker	1
Catering staff member	1

Observations and interviews were conducted over 3 months at each hospice (April–December 2021), including 26 observation visits to hospice 1 (109.5 h) and 27 to hospice 2 (126.5 h), during mornings, afternoons and evenings.

Ten interviews were carried out (five at each hospice) with five nurses, three doctors, one physiotherapist and one HCA.

### Thematic findings

The names of patient participants have been changed in reporting the findings.

#### Theme 1a: Delirium prevention as fundamental care, ‘I don’t think. . .they’ll think by doing this I won’t cause a patient to have delirium. . .they see that as part of good care’. (MoAs: Values; norms)

Clinicians carried out many behaviours that are preventative for delirium routinely including encouraging patients to eat and drink, bladder and bowel care and treating infections. Participants explained these tasks were mostly not carried out with the aim of delirium prevention, but as part of person-centred fundamental care which was highly valued in hospice culture,

I don’t think. . .they’ll think by doing this I won’t cause a patient to have delirium. . . just person-centred care. . . they see that as part of good care and what it looks like. (Interview, P20, nurse)

It’s part of the ethos of the hospice itself so if you haven’t done that probably you feel like you haven’t done your work properly that day. (Interview, P2, doctor)

For example, clinicians and volunteers used person-centred engagement, paying careful attention to patient’s needs and preferences to encourage them to eat and drink.

Observation: ‘A volunteer (P38) described a patient who was close to the end of life who she encouraged to eat bites of a bacon sandwich while she talked with him about his grandson. . . and whether his grandson would have a bacon sandwich before going to play football.’

Observation: ‘A nurse (P39) filled Christine’s beaker with more water. Christine’s speech was slurred and the nurse went round to the other side of the bed to get closer so she could make sure she heard what she said correctly. She gave her a choice of flavours of juice. Christine said she didn’t want to drink it yet, so the nurse left the bottle on the side table for later. She asked Christine if she wanted a hot drink and listened carefully, getting her to repeat herself, to make sure she had heard what drink she wanted correctly, coffee with sugar and no milk.’

Although many preventative behaviours were carried out as part of fundamental care, there were limitations due to lack of specific attention to delirium prevention and care. On admission, routine assessment of delirium risk factors, including medication, and routine screening for delirium, were not observed. This was not seen as part of fundamental, person-centred care delivery. One hospice had no delirium prevention documentation. In the other, a delirium care plan included risk factors but was only intended to be completed for patients with a known cognitive impairment or positive 4AT screen (not routinely used on admission). Therefore, patients’ risk factors were only recorded after they developed delirium symptoms, and this was often delayed or not completed.

Although clinicians engaged patients in conversation, providing some cognitive stimulation, they did not routinely orientate all patients to person, place and time. When patients were disorientated, aids, such as clocks or whiteboards, were used inconsistently.

Observation, doctors’ ward round: ‘Victor was unsure of the date. A doctor (P56) suggested writing the date on a whiteboard in his room and he agreed.’

Observation, doctors’ ward round (the following week): ‘The whiteboard still said the date of last week’s ward round. A doctor (P54) went to get a pen and changed the date.’

##### Theme 1b: Adequate staffing, multi-disciplinary team (MDT) engagement and role clarity support delirium prevention behaviours during fundamental care (MoAs: Environmental context and resources; norms; social/professional roles and identity)

Thorough fundamental care was supported by adequate staffing levels. Staff routinely spent time interacting with patients in a person-centred way, while meeting essential care needs. For example,

Observation: ‘09.30am: With Jill’s agreement, I went in with the nurse (P47) and Health Care Assistant (HCA; P60) while they gave her a wash in bed. The HCA knew Jill liked her false teeth to be put in cold water rather than tepid water before she put them in her mouth. It was her birthday today and the staff had hung her cards up on a string on the wall opposite her bed so she could see them. They chatted and joked with her while carrying out her care. They spent approximately 45 minutes with her’.

This was supported by higher hospice staffing levels, compared to hospital settings and a cultural norm encouraging staff to spend time on these activities with patients,

I think adequate staffing if we can, and I think we’re very lucky here. . .compared to hospital wards. . .staff don’t feel. . .rushed. . .we try and have this culture whereas. . . if somebody needs an hour and a half to have a bath or a shower then that’s fine. . .So it’s. . .like giving them permission to take a little bit longer if they feel as if they need to take a bit longer. (Interview, P20, nurse)

The value the MDT placed upon meeting essential care needs was reflected in, and supported by, their verbal and written communication. At both hospices, the admission assessment included many essential care needs relevant to delirium prevention including nutrition, bladder and bowel function, infection, breathlessness, sensory needs and pain assessment. These were monitored and followed up in nursing handovers, MDT meetings and clinical records.

All MDT members saw some preventative behaviours as part of their role. For example, although HCAs routinely went into patients’ rooms to offer them drinks, all members of the MDT offered patients drinks during other care tasks, ward rounds and therapy sessions. One doctor described demonstrating this is everybody’s role,

I try and. . . both role model but sort of instil. . .it’s a team effort; so. . . I’ll try and check with a patient before I leave, do they need anything right now, and if that’s a cup of tea we’ll go and get it for them, it’s not finding a nursing staff to say, oh so-and-so wants a cup of tea. . .I think. . .it’s everybody’s role. . . which is important. (Interview, P56, doctor)

The MDT worked together in complementary roles for many other preventative behaviours for example, nurses and HCAs monitoring pain, bladder and bowel function and signs of infection during usual care with the doctors leading investigations, treatment and medication review. However, when staffing resources were reduced, some preventative behaviours were carried out less thoroughly, due to role conflict. At one hospice, mealtime roles were clearly delineated: catering staff took meals to patients and HCAs supported them to eat. At the other, due to COVID restrictions, this role had shifted from volunteers to HCAs and nurses, with the increased workload described as difficult to manage,

Observation: ‘A nurse (P44) explained that the volunteers used to go round to patients with the breakfasts, lunches and teas. They used to wash up afterwards. Some volunteers could sit with patients and help them eat if they needed it, “or at least they would free us up to be able to do that” by doing the rest of the tasks. Now the HCAs and nurses have to do it all, “without any extra staff.”’

Similarly, although nursing staff assisted some patients to mobilise during usual care, at one hospice, therapy input was reduced. Conflicting views and safety concerns were expressed about increasing nurses’ role,

Observation, doctors’ ward round: ‘A doctor (P51) said the Occupational Therapist had been repeatedly asking the nurses. . . to mobilise patients whose needs were relatively straightforward. The doctor said that this wasn’t happening and that it was an issue in relation to improving patients’ mobility.’

Observation nursing handover: ‘A nurse (P50) said she was concerned that if the nurses started doing it, it will be another thing that is added to their role, ‘the nurses can do it’, and they will never get enough physio input’.

#### Theme 2: Limitations to delirium prevention in the context of hospice patients’ illness progression

##### Theme 2a: Patients’ reduced physical capability (COM-B) due to illness progression can limit delirium prevention behaviours

Symptoms including pain, fatigue and difficulty swallowing, which are common in cancer and other life-limiting illnesses, reduced patients’ ability to engage in behaviours preventing delirium including eating, drinking, mobilising and a regular sleep routine. Clinicians addressed these reduced capabilities by using beakers and syringes for fluids, soft food diets, flexible mealtimes and focusing on bed exercises when mobility was reduced. However, as patients neared the end of life, these influences became less modifiable.

##### Theme 2b: ‘Just want to wrap people up and care for them’: Clinicians’ norms of care limiting delirium prevention (MoA: Values, Norms)

It wasn’t only patients’ reduced physical capability during illness progression and close to the end of life that limited delirium prevention behaviours, but also clinicians’ expectations, values and norms related to this. Prioritising patient comfort was highly valued by clinicians,

The main thing is keeping the patients comfortable (Interview, P16, nurse)

Just trying to make the person as comfortable as they can. (Interview, P20, nurse)

Behavioural norms such as nursing staff wanting to, ‘wrap people up and care for them’ (Interview, P56, doctor) limited preventative behaviours, including encouraging patients to mobilise, eat and have a regular sleep routine. This may be appropriate close to the end of life, but therapists and doctors described it sometimes reducing patients’ functional independence,

Quite often people will come in and. . .the sort of automatic thing is to kinda wrap them up and, and look after them and quite often that means that they lose the ability to. . .be independent and doing certain things that they’ve been doing all the time they’ve been at home. . . so we have to make sure that we maintain that. (Interview, P63, Physiotherapist)

Some daytime sleep may be needed by patients experiencing fatigue. But nursing staff commonly left patients to sleep through the day with some unconcerned if this resulted in night-time waking,

Really with our patients if they are sleeping let them sleep; and then if they happen to be awake in the night, well there’s people around that can keep them company, make them drinks. . . (Interview, P47, nurse)

This doctor recognised that a disturbed sleep/wake routine could increase delirium risk,

The team. . .will let somebody sleep if they want to sleep, which is fine. . .the problem is when you then start to get disturbances in your days and nights, and we’ve seen that not uncommonly. . .with patients who develop a delirium. (Interview, P56, doctor)

However, regarding intervening to establish a regular sleep routine, ‘to try and help break that difficulty. . .we don’t push that very much’ (Interview, P56, doctor)

Daytime sleeping reduced opportunities to eat and mobilise, with patients commonly sleeping through mealtimes. They were sometimes offered a meal when they awoke, but this was not always the case.

Observation: ‘12.00 Lunchtime: Isaac wasn’t woken to have lunch and his meal was not left in his room. 1.30pm Nursing handover: Nurse handed over that he had slept a lot of the morning. Didn’t mention he had missed lunch.’

This doctor identified a need for clinicians to differentiate more between patients close to the end of life, and those with a better prognosis, who could be more actively encouraged to eat,

I think there’s some of those patients where we should be. . . more actively encouraging them. . . I think sometimes we don’t recognise as well patients who have got a better prognosis. . . cos, you know, I think if someone’s in their last weeks of life if they really don’t want to eat then that’s entirely their choice. (Interview, P56, doctor)

Delirium prevention could reduce patient distress, but this was rarely considered during decision-making aimed at patient comfort. When patients and their families made choices during usual care (e.g. whether to get up or have lunch), or regarding more ‘clinical’ interventions (e.g. artificial hydration), clinicians sometimes discussed benefits and disadvantages with them, but this rarely included delirium.

Doctors and nursing staff did consider the potential for medications to make patients drowsy or ‘muddled’, but commonly conceptualised these as ‘side effects’ rather than delirium symptoms. Doctors commonly discussed this ‘trade-off’ between pain relief and sleepiness or confusion with patients, and followed their preferences,

Sometimes I think people are very good at being able to say to you. . . if this makes me a little bit more sleepy I don’t mind because this pain’s so bad that. . . I’ll trade it for that, whereas we’ll equally have patients who’ll be like. . . I’d like to have my pain controlled but you can’t make me drowsy, you can’t make me a little bit muddled with things, I need to be sharp cos I’m still doing this. (Interview, P1, doctor)

When patients appeared distressed but were unable to communicate the cause, doctors sometimes tried multiple medications, with the aim of patient comfort,

So, in some cases then we tend to treat all the likely causes if we can’t identify one specific cause. So, for example, if somebody is quite agitated you may use both an, anxiolytic and pain relief at the same time or you may want to use any sequence and say, OK, because of pain, I can’t really tell and you try pain relief and see if that settle the person and if it doesn’t then say, OK, let’s try the anxiolytic. . . like Midazolam, or antipsychotics sometimes like Haloperidol. (Interview, P2, doctor)

This may have the unintended consequence of causing or increasing delirium and related distress.

## Discussion

### Main findings

Clinicians carried out many preventative behaviours as part of person-centred fundamental care, without delirium prevention as an explicit aim. Carrying out essential care tasks was highly valued and supported by adequate staffing levels, MDT engagement and role clarity.

In the in-patient hospice setting, patients’ reduced physical capability limited some delirium prevention behaviours, as did clinicians’ behavioural norms, including nursing staff ‘wrapping patients up’ to care for them. These may be appropriate when patients are close to the end of life but doctors and therapists highlighted that there may be a need to differentiate more between patients who are earlier in their illness trajectory, for whom a more active approach to care may be beneficial. Delirium prevention was not embedded into routine assessment and care-decision-making, despite its potential to reduce patient distress.

### Study strengths and limitations

We used focused ethnography which enabled observation of fundamental care behaviours relevant to delirium prevention.^23^ The use of behaviour change theory enabled systematic analysis of behavioural influences which can be targeted for intervention using behaviour change techniques.^20^ It also enhances transferability, enabling comparison with other delirium prevention studies using the same model.^40^ The use of ethnographic methods with this behaviour change approach is a novel methodological development.^37,41^ A limitation of this study is that some COVID-19-related restrictions remained in place during the observation period which will have affected practice observed, particularly in relation to person-centred communication and reduced family and volunteer involvement.

### What this study adds?

Many behaviours that contribute to preventing delirium should be carried out as part of fundamental care^42^ but in hospitals, these tasks are commonly under-valued and carried out inconsistently.^8,10^ In contrast, in the in-patient hospice setting, we found fundamental care was highly valued and, consequently, many delirium preventative behaviours were carried out consistently. Similarly, in Australian palliative care units a compassionate care culture was found to support delirium prevention.^40^ Other settings could learn valuable lessons from specialist palliative care in this regard.

Although many preventative behaviours were carried out during fundamental care, there were limitations due to lack of specific attention to delirium prevention and care. Clinicians did not routinely re-orientate all patients. Routine assessment of delirium risk factors on admission, and routine delirium screening, as recommended in guidelines,^31,32^ was not implemented. This was not seen as part of fundamental, person-centred care delivery. Clinicians may anticipate this would be too burdensome for patients,^43^ but doctors’ review of existing clinical data for risk factors that could be modified without intrusive treatment could be beneficial (e.g. constipation and medication).

In hospitals, high workloads influence poor delivery of fundamental care and delirium prevention interventions.^44^ We found higher staffing levels and cultural acceptability of spending time carrying out person-centred essential care, supported delirium prevention behaviours. This reflects the value placed upon fundamental care at an organisational level.

Hospital-based studies report that essential care is commonly viewed as HCAs’ role^8,45,46^ and tasks seen as everyone’s responsibility are poorly completed.^47^ In contrast, in the hospice setting, we found multidisciplinary collaboration and engagement, including senior clinicians role-modelling expected behaviours, supported delirium prevention, in line with recommendations for promoting team ‘buy in’.^10,44,48^ However, when staffing resources were reduced, there was a need for explicit negotiation of role expectations, to ensure agreement, training and resolution of risk-related concerns, which have similarly been identified as a barrier in other studies.^47,49^

There are limitations to delirium prevention in hospices due to patients’ illness severity, symptoms of pain and fatigue and unacceptability of more intrusive interventions.^43^ However, some limiting factors could potentially be modified. Behavioural norms related to prioritising patient comfort in palliative care^50^ limited preventative behaviours. Similarly, a cultural norm of ‘tucking up’ patients has been identified as a barrier to ‘rehabilitative’ palliative care which aims to preserve independence for as long as possible.^51,52^ We are not proposing changing the underlying value of patient comfort, but rather, how clinicians’ behavioural norms could be developed, to achieve it more fully.^53^ As delirium prevention has the potential to reduce patient distress, it is relevant to care decision-making aimed at patient comfort, but was rarely included. Our previous interview study^16^ recommended developing team understanding of the role of delirium prevention in reducing distress. This study also identified the need to increase understanding, particularly for nurses and health care assistants, of the benefit of continuing preventative behaviours as patients’ illness progresses for example, encouraging eating and mobilising. Green et al.^40^ suggest staff’s motivation to enable these behaviours later in patients’ illness trajectories, may increase with better understanding of its link with delirium prevention.

As described in previous studies, medications associated with delirium risk were frequently used with the aim of patient comfort.^54–56^ Sleepiness and some confusion, were accepted as a trade-off for pain control. Re-framing doctors’ and nursing staff’s understanding of these ‘side effects’ as possible delirium symptoms could enhance medication review. Valuable learning may be gained from ICU, in which long-held practice norms of deep sedation have been challenged through interventions reducing delirium risk by enabling patients to be more awake, cognitively engaged and physically active.^15^

Our findings suggest that many delirium prevention behaviours are carried out as part of fundamental care in hospices. A behaviour change intervention should focus on those aspects that could be improved:

- Prioritisation of specific delirium prevention behaviours: Routine doctors’ review of delirium risk factors on admission, including medication; addressing patients’ cognitive needs so that these are incorporated as part of fundamental, person-centred care delivery.
- Increasing clinicians’ understanding of the benefit of delirium prevention behaviours during patients’ illness progression to reduce distress.^20^
- Changing staff behavioural norms to enable continuation of preventative behaviours when possible.

This could be delivered through case study-based reflective group learning with further sessions for clinician feedback and reflection on putting this approach into practice. Nursing and therapy leaders, as well as people with lived experience of delirium, could be involved in intervention delivery.^20^

Important implications of our study findings for patients and caregivers include that delirium prevention has the potential to reduce patient distress and there is a need to include it in care decision-making discussions with patients and families.

Through our study, we have identified delirium prevention behaviours, and influences upon them, that could be targeted in an intervention for the inpatient hospice setting. Next steps should involve further stakeholder consultation regarding the modifiability of these behaviours and the feasibility and acceptability of the strategies proposed. Further studies to develop effective interventions for both delirium prevention and management are needed in palliative care contexts.

## Supplemental Material

sj-docx-1-pmj-10.1177_02692163241310762 – Supplemental material for Delirium prevention in hospices: Opportunities and limitations – A focused ethnography Supplemental material, sj-docx-1-pmj-10.1177_02692163241310762 for Delirium prevention in hospices: Opportunities and limitations – A focused ethnography by Imogen Featherstone, Miriam J Johnson, Trevor Sheldon, Rachael Kelley, Rebecca Hawkins, Alison Bravington, Sarah Callin, Rachael Dixon, George Obita and Najma Siddiqi in Palliative Medicine

sj-docx-2-pmj-10.1177_02692163241310762 – Supplemental material for Delirium prevention in hospices: Opportunities and limitations – A focused ethnography Supplemental material, sj-docx-2-pmj-10.1177_02692163241310762 for Delirium prevention in hospices: Opportunities and limitations – A focused ethnography by Imogen Featherstone, Miriam J Johnson, Trevor Sheldon, Rachael Kelley, Rebecca Hawkins, Alison Bravington, Sarah Callin, Rachael Dixon, George Obita and Najma Siddiqi in Palliative Medicine

sj-docx-3-pmj-10.1177_02692163241310762 – Supplemental material for Delirium prevention in hospices: Opportunities and limitations – A focused ethnography Supplemental material, sj-docx-3-pmj-10.1177_02692163241310762 for Delirium prevention in hospices: Opportunities and limitations – A focused ethnography by Imogen Featherstone, Miriam J Johnson, Trevor Sheldon, Rachael Kelley, Rebecca Hawkins, Alison Bravington, Sarah Callin, Rachael Dixon, George Obita and Najma Siddiqi in Palliative Medicine
