# Supplementary material for: Delirium prevention in hospices: Opportunities and limitations – A focused ethnography
Source: Palliat Med. 2025 Jan 21;39(3):391–400. doi: 10.1177/02692163241310762 (PMC11877983; doi:10.1177/02692163241310762)
Supplement: sj-docx-1-pmj-10.1177_02692163241310762 – Supplemental material for Delirium prevention in hospices: Opportunities and limitations – A focused ethnography [file sj-docx-1-pmj-10.1177_02692163241310762.docx]

**Supplementary file 1: Definitions of Behaviour Change Theory Concepts**

**Definitions of COM-B components**

| **COM-B component** | **Definition** |
| --- | --- |
| Physical capability | Physical skill, strength or stamina |
| Psychological capability | Knowledge or psychological skills, strength or stamina to engage in the necessary mental processes |
| Physical opportunity | Opportunity afforded by the environment involving time, resources, locations, cues, physical ‘affordance’ |
| Social opportunity | Opportunity afforded by interpersonal influences, social cues and cultural norms that influence the way we think about things. |
| Reflective motivation | Reflective processes involving plans (self-conscious intentions) and evaluations (beliefs about what is good and bad) |
| Automatic motivation | Automatic processes involving emotional reactions, desires (wants and needs), impulses, inhibitions, drive states and reflex responses. |

Michie et al (2011)

**Definitions of Mechanisms of Action**

**Attitude towards the Behaviour:** The general evaluations of the behaviour on a scale ranging from negative to positive.

**Behavioural Cueing:** Processes by which behaviour is triggered from either the external environment, the performance of another behaviour, or from ideas appearing in consciousness.

**Behavioural Regulation:** Behavioural, cognitive, and/or emotional skills for managing or changing behaviour.

**Beliefs about Capabilities:** Beliefs about one’s ability to successfully carry out a behaviour

**Beliefs about Consequences:** Beliefs about the consequences of a behaviour (i.e., perceptions about what will be achieved and/ or lost by undertaking a behaviour, as well as the probability that a behaviour will lead to a specific outcome).

**Environmental Context and Resources:** Aspects of a person’s situation or environment that discourage or encourage the behaviour.

**Feedback Processes:** Processes through which current behaviour is compared against a particular standard.

**General Attitudes/Beliefs:** Evaluations of an object, person, group, issue, or concept on a scale ranging from negative to positive.

**Goals:** Mental representations of outcomes or end states that an individual wants to achieve.

**Intentions:** A conscious decision to perform a behaviour or a resolve to act in a certain way.

**Knowledge:** An awareness of the existence of something.

**Memory, Attention, and Decision Processes:** Ability to retain information, focus on aspects of the environment, and choose between two or more alternatives.

**Motivation:** Processes relating to the impetus that gives purpose or direction to behaviour and operates at a conscious or unconscious level.

**Needs:** Deficit of something required for survival, well-being, or personal fulfilment.

**Norms:** The attitudes held and behaviours exhibited by other people within a social group.

**Optimism:** Confidence that things will happen for the best or that desired goals will be attained.

**Perceived Susceptibility/Vulnerability:** Perceptions of the likelihood that one is vulnerable to a threat.

**Reinforcement:** Processes by which the frequency or probability of a response is increased through a dependent relationship or contingency with a stimulus or circumstance.

**Self-image:** One’s conception and evaluation of oneself, including psychological and physical characteristics, qualities, and skills.

**Skills:** An ability or proficiency acquired through practice.

**Social Influences:** Those interpersonal processes that can cause oneself to change one’s thoughts, feelings, or behaviours.

**Emotion:** A complex reaction pattern involving experiential, behavioural, and physiological elements.

**Social Learning/Imitation:** A process by which thoughts, feelings, and motivational states observed in others are internalized and replicated without the need for conscious awareness.

**Social/Professional Role and Identity:** A coherent set of behaviours and displayed personal qualities of an individual in a social or work setting.

**Subjective Norms:** One’s perceptions of what most other people within a social group believe and do.

**Values:** Moral, social or aesthetic principles accepted by an individual or society as a guide to what is good, desirable, or important.

Connell et al (2019)
