# Supplementary material for: Delirium prevention in hospices: Opportunities and limitations – A focused ethnography
Source: Palliat Med. 2025 Jan 21;39(3):391–400. doi: 10.1177/02692163241310762 (PMC11877983; doi:10.1177/02692163241310762)
Supplement: sj-docx-2-pmj-10.1177_02692163241310762 – Supplemental material for Delirium prevention in hospices: Opportunities and limitations – A focused ethnography [file sj-docx-2-pmj-10.1177_02692163241310762.docx]

**Supplementary File 2: Observation guide**

(Please note: Observation of delirium recognition, risk reduction and management was conducted during this study but only delirium prevention (risk reduction) findings are reported in this paper so only the relevant sections of the observation guide are included here.)

**Observation guide overview**

**Stages of delirium care**

**Recognition**

**-**Change in behaviour/symptoms ax/ recognised? Not?

- Family involvement?

-Screening/ diagnosis?

-Team communication?

-Terminology?

**Risk reduction**

**-**Ax of clinical factors?

Behaviours in relation to:

-Orientation -Sensory needs

-Sleep -Mobility

-Pain -Hydration/nutrition

-Bladder/ bowel function

-Hypoxia - Infection

-Medication

**Management**

-Investigation/ treatment of underlying cause(s)?

-Person-centred care/ communication

-Family involvement/ support

-Managing agitation/ risk

-Medication-type? purpose?

**COM-B**

**How are the following influencing this behaviour?**

**Capability:**

Understanding/ skill?

Physical capability?

**Motivation:**

Emotions?

Habits/ routines?

Belief in importance?

Goals/ plans?

**Opportunity:**

Time/ resources?

Environment?

Cultural norms?

Social influences?

WHAT is the behaviour? (relevant to delirium care)

What is not being done?

WHO is doing the behaviour? (staff role)

Are other people involved? (team, family)

WHEN? How routinely is it being done?

WHY? Is it intended as part of delirium care?

Are there hospice/ palliative care specific influences on this behaviour?

Impact of COVID-19 situation on the behaviour?

**DELIRIUM RISK REDUCTION**

WHO? Staff role; family involvement; team communication?

WHEN? How routinely?

WHY? Is it intended as part of delirium care? Understanding of delirium risk reduction.

- Ax of clinical factors that could contribute to delirium? Within 24 hours of admission?
- MCI? (tailored to individual risk factors, reversibility, patient’s goals of care, setting).
- **Orientation/ addressing cognitive impairment:**
- Interacting/ talking?(e.g. where they are, who you are)
- Cognitively stimulating activities? (e.g. reminiscence)
- Orientation aids? (e.g. clock, calendar, board with staff names/ date)
- Familiar possessions?
- Room changes? Continuity of care providers?
- **Sensory needs:**
- Glasses/ hearing aids?
- Reversible causes (e.g. impacted ear wax)
- Environment- light, noise, activity?
- **Sleep:**
- Non-pharma approaches to aid night-time sleep? E.g. relaxing music, lighting, non-caffeine drinks.
- Minimise noise/ clinical activities during sleep periods?
- Minimise use of sedative hypnotics?
- **Mobility** (within ability/ energy limits)**:**
- Encourage to walk if able (provide walking aids), carry out selfcare/ valued activities, active range of motion exercises?
- Physio/ OT ax/ input?
- Minimise use of equipment that can restrict movement? e.g. intravenous lines, indwelling catheters.
- **Pain management**
- Ax and monitor for pain, including non-verbal signs?
- Use of non-pharma approaches?
- Use of pain meds: Paracetamol for mild pain? Opioids- minimum effective dose? Adjunct therapies to minimise dose?
- **Hydration/ nutrition**
- Encourage/ assist drinking? (Issues re. difficulty swallowing? Consciousness?)
- If dehydrated- consider use of subcut/ intravenous fluids? (patient’s goals of care, prognosis, burden of treatment, likely efficacy?)
- How address nutritional needs?
- Dietician input?
- **Bladder/ bowel function**
- Monitor?
- Strategies to promote normal elimination pattern?
- Strategies to prevent urinary retention/ constipation?
- **Hypoxia**
- Ax for hypoxia?
- Optimise oxygen sats? (if clinically appropriate)
- **Infection**
- Infection control procedures?
- Look for/ treat infection?
- Avoid unnecessary catheterisation?
- **Medication review:**
- Number, changes, medications with high risk for delirium? e.g. anticholinergics, psychoactive medications, benzodiazapines, opiates.
- De-prescribe to reduce polypharmacy.
- Withdraw meds that are high risk for delirium if possible, or use lowest possible dose or substitute with similar, lower risk medication.
