# Supplementary material for: Delirium prevention in hospices: Opportunities and limitations – A focused ethnography
Source: Palliat Med. 2025 Jan 21;39(3):391–400. doi: 10.1177/02692163241310762 (PMC11877983; doi:10.1177/02692163241310762)
Supplement: sj-docx-3-pmj-10.1177_02692163241310762 – Supplemental material for Delirium prevention in hospices: Opportunities and limitations – A focused ethnography [file sj-docx-3-pmj-10.1177_02692163241310762.docx]

**Supplementary file 3: Table of influences on delirium prevention behaviour: Optimise mobility**

| **Cross-cutting themes/ subthemes the influences relate to** | **Influence: summary statement** | **MoA(s)** | **COM-B** |
| --- | --- | --- | --- |
| **1a. Delirium prevention as fundamental care** | Staff use person-centred interpersonal skills to encourage patients to mobilise | Skills, Values | Psychological capability, reflective motivation |
| **1b. Adequate staffing, MDT engagement and role clarity** | Physiotherapist’s specialist input | Skills | Psychological and physical capability |
|  | Physiotherapy time/ staffing levels | Environmental context and resources | Physical opportunity |
|  | Nurses and HCAs assist some patients to mobilise during usual care in liaison with physio | Skills | Physical and psychological capability |
|  | Lack of clarity regarding physio/ nurses/ HCAs roles in mobilising patients | Social/ Professional role and identity | Reflective motivation |
|  | MDT documentation of patients’ mobility needs supports staff to mobilise patients | Environmental context and resources | Physical and social opportunity |
|  | Verbal team communication of patients’ mobility needs supports staff to mobilise patients | Environmental context and resources | Social opportunity |
| **2a.Patients’ reduced physical capability limits delirium prevention behaviours** | The physical effects of patients’ medical conditions can reduce their ability to mobilise | x | Physical capability |
|  | When patients are sleepy or fatigued this can reduce their ability and motivation to mobilise | Motivation | Physical capability/ motivation |
|  | Patients’ pain can reduce their ability and motivation to mobilise | Motivation | Physical capability/ motivation |
|  | When patients are ‘deconditioned’ this can reduce their ability to mobilise |  |  |
|  | **Strategies used by staff to address patients’ physical limitations regarding mobility:** | | |
|  | Flexible problem-solving to manage patients’ fatigue can support them to mobilise |  |  |
|  |  | Memory, attention and decision processes | Psychological capability |
|  | Pharmacological pain management may sometimes support patients to mobilise | Skills | Physical and psychological capability |
|  | Non-pharma pain management may sometimes support patients to mobilise | Skills | Physical and psychological capability |
|  | Provision of suitable equipment can support patients to mobilise | Skills |  |
|  | Physiotherapists adapt their input to patients’ physical capabilities | Memory, attention and decision processes | Psychological capability |
|  | Staff use a graded approach to support patients to mobilise | Skills | Psychological capability |
| **2b. Clinicians’ norms of care limiting delirium prevention** | Staff’s expectations of patients’ mobility goals are different in the hospice setting than in hospital/ rehabilitation settings | Goals, Norms | Reflective motivation, Social opportunity |
|  | Staff’s perception of the patient’s stage of illness influences their expectations regarding the patient’s mobility | Norms | Reflective motivation, Social opportunity |
|  | Staff follow patients’ preferences regarding whether they want to mobilise | Values | Reflective motivation |
| **Behaviour-specific influences** |  | | |
| Patient safety/ risk of falls | Some patients’ fear of falling is a barrier to them mobilising | Perceived susceptibility/ vulnerability | Reflective motivation |
|  | Nurses and HCAs are concerned about confused patients’ risk of falls | Perceived susceptibility/ vulnerability (patient) | Reflective motivation |
|  | Nurses and HCAs feel anxious about patients’ risk of falls | Emotion | Automatic motivation |
|  | Supporting confused patients to mobilise safely/ reduce their risk of falls is time and staff intensive | Environmental context and resources | Physical opportunity |
|  | Some nurses feel they don’t have the skills to mobilise some patients safely | Skills | Physical and psychological capability |
|  | Some nurses are concerned they may be blamed if a patient falls when they are supporting them to mobilise | Perceived susceptibility/ vulnerability (staff) | Reflective motivation |
| Psychological factors can reduce patients motivation to mobilise | Low mood can reduce patients motivation to mobilise | Motivation | Reflective motivation |
|  | Embarrassment at using walking aids can reduce patients ability to mobilise | Self-image | Reflective motivation |
| Environmental factors | Confused patients opportunity to ‘wander’ is limited by the hospice environment | Environmental context and resources | Physical opportunity |
